# Opsoclonus-myoclonus syndrome associated with pancreatic neuroendocrine tumor: a case report

**DOI:** 10.1186/s12883-022-03012-6

**Published:** 2022-12-30

**Authors:** Raphael Reinecke, Annemarie Reiländer, Alexander Seiler, Christine Koch, Martin Voss

**Affiliations:** 1 Department of Neurology, Goethe University Frankfurt, Frankfurt am Main, Germany; 2 Dr. Senckenberg Institute of Neurooncology, Goethe University Frankfurt, Frankfurt am Main, Germany; 3 Department of Gastroenterology, Hepatology and Endocrinology, Goethe University Frankfurt, Frankfurt am Main, Germany

**Keywords:** Opsoclonus-myoclonus syndrome, Pancreatic neuroendocrine tumor, Paraneoplastic neurological syndromes, Plasmapheresis

## Abstract

**Background:**

Opsoclonus-myoclonus syndrome (OMS) is a rare, immune-mediated neurological disorder. In adults, the pathogenesis can be idiopathic, post-infectious or paraneoplastic, the latter etiology belonging to the ever-expanding group of defined paraneoplastic neurological syndromes (PNS). In contrast to other phenotypes of PNS, OMS cannot be ascribed to a single pathogenic autoantibody. Here, we report the first detailed case of paraneoplastic, antibody-negative OMS occurring in association with a pancreatic neuroendocrine tumor (pNET).

**Case presentation:**

A 33-year-old female presented with a two-week history of severe ataxia of stance and gait, dysarthria, head tremor, myoclonus of the extremities and opsoclonus. Her past medical history was notable for a metastatic pancreatic neuroendocrine tumor, and she was subsequently diagnosed with paraneoplastic opsoclonus-myoclonus syndrome. Further workup did not reveal a paraneoplastic autoantibody. She responded well to plasmapheresis, as she was refractory to the first-line therapy with corticosteroids.

**Conclusions:**

This case expands current knowledge on tumors associated with paraneoplastic opsoclonus-myoclonus syndrome and the age group in which it can occur. It further adds evidence to the effectiveness of plasmapheresis in severe cases of opsoclonus-myoclonus syndrome with a lack of response to first-line therapy.

**Supplementary Information:**

The online version contains supplementary material available at 10.1186/s12883-022-03012-6.

## Background

Opsoclonus-myoclonus syndrome (OMS) is a rare disorder of autoimmune or paraneoplastic origin. Often described as ‘dancing eyes and feet’ syndrome, it consists of a combination of opsoclonus and limb and trunk dominant myoclonus, further associated with balance difficulties, ataxia and tremor [1]. In children, the syndrome has a paraneoplastic etiology in about half of cases and is frequently associated with neuroblastoma. The remaining cases are idiopathic or assumed to have a postviral etiology [2]. In adults, paraneoplastic (P-OMS) and idiopathic (I-OMS) cases differ in age, with idiopathic cases occurring at a younger age on average (mean age 40 vs. 55 years) [3]. Except for ovarian teratoma, in most cases of OMS under the age of 40 years, the etiology is idiopathic or post-infectious, both of which tend to have a better prognosis in comparison to children and P-OMS [4]. A paraneoplastic association is found in 20–40% of all cases. Commonly associated malignancies are small cell lung cancer and adenocarcinoma of the breast, but other tumors such as ovarian teratoma, gastric and pancreatic adenocarcinoma and malignant melanoma have also been reported [5–7]. Most adults will not test positive for established autoantibodies. In single cases, mostly anti-Ri/-Anna2 antibodies in association with breast cancer and glycine receptor antibodies in association with lung cancer are reported [3]. Recently, novel autoantibodies have been described in relation to cases of OMS, amongst them HNK-1 cell surface epitope and Kelch-like protein-11 antibodies [3, 8].

Here, we discuss the case of a young female patient with paraneoplastic OMS in association with a pancreatic neuroendocrine tumor (pNET), a not yet described tumor association, with a remarkable therapeutic response to plasmapheresis.

## Case report

The 33-year-old female patient was referred to our hospital for two weeks of gait abnormalities and slurred speech, accompanied by nausea and vomiting.

Her past medical history was notable for a moderately differentiated (G2) pNET (Ki67 ratio of 15%) with pulmonary, hepatic and lymphogenic metastases, which was initially diagnosed ten months before presentation. She was treated with five cycles of 5-fluorouracil (FU) and streptozotocin until one month before presentation and was switched to long-acting octreotide right before the onset of her symptoms.

Neurologic examination revealed severe ataxia of stance and gait, dysarthria, head tremor, myoclonus of all extremities and the typical involuntary, high-frequency, chaotic multidirectional saccadic eye movements of opsoclonus. A clinical diagnosis of a complete opsoclonus-myoclonus syndrome (OMS) was established. The modified Rankin Scale (mRS) at symptom onset was 4.

A contrast-enhanced MRI of the brain was unremarkable, without sign of brain or leptomeningeal metastasis. Cerebrospinal fluid (CSF) examination revealed a pleocytosis of 12 leukocytes /µl (< 5 /µl) and an elevated lactate of 2.6 mmol/l (0.6–2.1 mmol/l). Four oligoclonal bands restricted to the CSF were detected (type 2). CSF cytology revealed a lymphocytic predominance without the detection of atypical cells. CSF culture and a multiplex polymerase chain reaction (PCR) assay for the detection of typical meningitis pathogens were negative. A comprehensive antibody panel with an immunofluorescence test (IFT) and a cell-based assay (CBA) (Euroimmun, Lübeck, Germany) was negative for antineuronal and paraneoplastic antibodies (see Supplement 1 for a complete list). Serum studies for HIV and hepatitis viruses were negative without evidence of active or prior infection. CT imaging of the chest and abdomen revealed a stable disease without any evidence for tumor progression.

The patient was treated with methylprednisolone (1.0 g) for five consecutive days, followed by a slow steroid taper. There was only minimal improvement in symptoms one week after steroid pulse therapy; thus, therapy was switched to plasmapheresis with an accompanying slow prednisolone taper, starting at 70 mg daily. The patient received a total of eight cycles of plasmapheresis, during which there was significant improvement in symptoms. Opsoclonus and tremor regressed completely after two weeks. She was able to sit unassisted but was not yet able to walk or stand on her own. Tumor therapy was switched to capecitabine and dacarbazine since the P-OMS was considered a clinical progression in the absence of clear radiological progression.

**Fig. 1:**
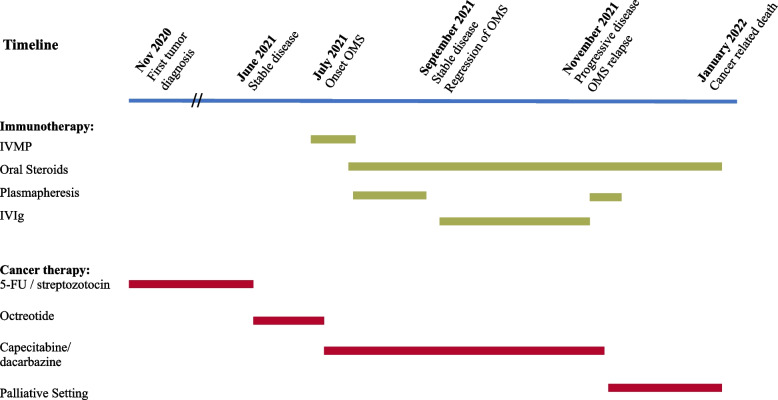
Disease course and timeline of immunotherapy and cancer treatment. OMS, opsoclonus-myoclonus syndrome; IVMP, high-dose intravenous methylprednisolone; IVIg, intravenous immunoglobulins; 5-FU, 5-fluorouracil

At her two-month follow-up, the patient continued to be clinically stable without a renewed worsening of symptoms. The patient continued to require assistance when walking due to ataxia but otherwise had complete regression of all other symptoms (mRS = 3). Maintenance therapy with IVIgs was initiated and given every four weeks. Four months after the onset of OMS, the patient had significant tumor progression under the existing immunotherapy, which, in addition to marked nausea and cholestasis, worsened her neurological symptoms. Plasmapheresis was re-initiated but stopped after five cycles at the patient's request due to her poor general condition. The patient underwent debulking surgery for general symptom control in a palliative setting (see Fig. 1 for an overview of the course of the disease and the therapy that has been performed).

## Discussion and conclusions

OMS belongs to the group of rare, immune-mediated neurologic manifestations of systemic tumors, summarized under the term paraneoplastic neurological syndromes (PNS), which develop in approximately 1 in 300 patients with cancer [9]. While the most common manifestations of PNS are limbic encephalitis (31%), cerebellar degeneration (28%) and encephalomyelitis (20%), P-OMS is significantly less frequent, with only 1% of PNS cases in a recent, representative population-based epidemiological study [9]. To account for the expanding field and detection of PNS, Graus et al. proposed a new systematization of symptoms, dividing them into ‘high-risk’ and ‘intermediate-risk’ phenotypes, and the associated antibodies into ‘high-risk’ and ‘intermediate-risk’, depending on their association with cancer (> 70% and 30–70%, respectively) [10]. Based on these characteristics, the newly introduced PNS-Care score divides the syndromes into definite, probable and possible PNS, depending on the phenotype, detected antibody and detection of a tumor on further workup [10]. Of note, among all PNS syndromes, only OMS does not require the detection of an associated antibody for the diagnosis of definite PNS, accounting for the rarity of antibodies found in OMS cases in the current literature [3].

In this patient, we describe the first case of OMS occurring in association with a pNET at an unusually young age. While paraneoplastic syndromes are a well-described complication of neuroendocrine small cell lung cancers, especially anti-Hu associated sensory neuropathy, cerebellar ataxia and encephalomyelitis [11], only a few PNS cases are reported in patients with pNET. Among these are a case of acute cerebellar dysfunction and spastic paraparesis with anti-Ri antibodies [12], an anti-GAD associated encephalomyelitis [13] and an anti-NMDA receptor encephalitis [14].

While on average, most patients with P-OMS present in patients over 50 years old, our patient was only 33 years old. In patients under 40 years of age, the most likely cause of OMS is idiopathic, post-infectious, or, in women, paraneoplastic and associated with ovarian teratoma [3, 15]. This case highlights the importance of performing a comprehensive tumor screening in patients at a younger age when OMS is present, according to the novel recommendations of the PNS care panel [10]. Had our patient’s tumor been unknown, her diagnosis could have been missed with only targeted screening for a teratoma.

Treatment of P-OMS is still mostly based on expert opinion due to the lack of prospective studies. In P-OMS, besides treatment of the underlying malignancy, first-line therapy consists of corticosteroids, intravenous immunoglobulins and plasmapheresis, similar to the treatment of autoimmune encephalitis [16]. Of note, our patient did not respond to high dose corticosteroids, but subsequently had a good therapeutic response to plasmapheresis. While systematic evidence is lacking, this case demonstrates the efficacy of plasmapheresis in steroid-refractory cases of P-OMS even in the absence of defined antibodies.

If a palliative setting had not been established, rituximab or cyclophosphamide would have been an option for second-line therapy. While the benefit of these two drugs alone or in combination has been shown in children with OMS for therapy escalation [17], no data exist for adult-onset OMS, and direct translation of the results is difficult.

Our case expands current knowledge on tumors associated with P-OMS and the age group in which it can occur, thus highlighting the importance of a comprehensive tumor evaluation in younger patients. It further adds evidence to the effectiveness of plasmapheresis in severe cases of OMS with a lack of response to first-line therapy.

## Supplementary Information

**Additional file 1.** 

## Data Availability

The data used and analyzed during the current study are available from the corresponding author on request.

## Abbreviations

OMS: Opsoclonus-myoclonus syndrome
P-OMS: Paraneoplastic opsoclonus-myoclonus syndrome
I-OMS: Idiopathic opsoclonus-myoclonus syndrome
pNET: Pancreatic neuroendocrine tumor
PNS: Paraneoplastic neurological syndromes
anti-Ri/-Anna2: Anti-neuronal nuclear antibody type 2
HNK-1: Human natural killer 1
Ki67: Marker of proliferation Ki-67
NET: Neuroendocrine tumor
CSF: Cerebrospinal fluid
MRI: Magnetic resonance imaging
PCR: Polymerase chain reaction
IFT: Immunofluorescence test
CBA: Cell based assay
HIV: Human immunodeficiency virus
CT: Computed tomography
anti-Hu: Anti-Hu antibody
anti-GAD: Anti-glutamate decarboxylase antibody
anti-NMDA: Anti-N-methyl-D-aspartate receptor antibody
