# Supplementary material for: Opsoclonus-myoclonus syndrome associated with pancreatic neuroendocrine tumor: a case report
Source: BMC Neurol. 2022 Dec 30;22:507. doi: 10.1186/s12883-022-03012-6 (PMC9801616; doi:10.1186/s12883-022-03012-6)
Supplement: Supplementary file 1 — Additional file 1. [file 12883_2022_3012_MOESM1_ESM.docx]

**Supplement 1:**

List of tested antibodies in serum and CSF: GAD65, Hu, Ri, Anna-3, Yo, Tr/DNER, Ma/Ta, Amphiphysin, Aquaporin-4, MOG, Glutamat-receptor (AMPA, NMDA), GABA-b-receptor, LGI1, CASPR2, IgLON5, ZIC4, DPPx, Anti-Myelin, CARPVIII, Glycin-receptor, mGluR1, mGluR5, GABA-a-receptor, Rho GTPase activating protein 26, Recoverin, GluRD2, Flotilin-1/2, ITPR1, Homer 3, Neurochondrin, Neurexin-3-alpha, ERC1, Sez6l2, AP3B2, Contactin1, Neurofascin 155, Neurofascin 186, AT1A3, KCNA2, Dopamin-receptor 2
